# Enhancement of Photoacoustic Signal Strength with Continuous Wave Optical Pre-Illumination: A Non-Invasive Technique

**DOI:** 10.3390/s21041190

**Published:** 2021-02-08

**Authors:** Anjali Thomas, Souradip Paul, Joy Mitra, Mayanglambam Suheshkumar Singh

**Affiliations:** 1 Biomedical Instrumentation and Imaging Laboratory (BIIL), School of Physics (SoP), Indian Institute of Science Education and Research Thiruvananthapuram (IISER-TVM), Thiruvananthapuram 695551, India; anjalithomas16@iisertvm.ac.in (A.T.); souradip.rkm16@iisertvm.ac.in (S.P.); 2 Scanning Probe Microscopy and Plasmonics Lab, School of Physics (SoP), Indian Institute of Science Education and Research Thiruvananthapuram (IISER-TVM), Thiruvananthapuram 695551, India; j.mitra@iisertvm.ac.in

**Keywords:** photoacoustic imaging, signal enhancement, pre-illumination, photo-thermal effect, heat capacity

## Abstract

Use of portable and affordable pulse light sources (light emitting diodes (LED) and laser diodes) for tissue illumination offers an opportunity to accelerate the clinical translation of photoacoustic imaging (PAI) technology. However, imaging depth in this case is limited because of low output (optical) power of these light sources. In this work, we developed a noninvasive technique for enhancing strength (amplitude) of photoacoustic (PA) signal. This is a photothermal-based technique in which a continuous wave (CW) optical beam, in addition to short-pulse $\left(\sim \;\mathrm{nsec}\right)$ laser beam, is employed to irradiate and, thus, raise the temperature of sample material selectively over a pre-specified region of interest (we call the process as pre-illumination). The increase in temperature, in turn enhances the PA-signal strength. Experiments were conducted in methylene blue, which is one of the commonly used contrast agents in laboratory research studies, to validate change in temperature and subsequent enhancement of PA-signal strength for the following cases: (1) concentration or optical absorption coefficient of sample, (2) optical power of CW-optical beam, and (3) time duration of pre-illumination. A theoretical hypothesis, being validated by numerical simulation, is presented. To validate the proposed technique for clinical and/or pre-clinical applications (diagnosis and treatments of cancer, pressure ulcers, and minimally invasive procedures including vascular access and fetal surgery), experiments were conducted in tissue-mimicking Agar phantom and ex-vivo animal tissue (chicken breast). Results demonstrate that pre-illumination significantly enhances PA-signal strength (up to ~70% (methylene blue), ~48% (Agar phantom), and ~40% (chicken tissue)). The proposed technique addresses one of the primary challenges in the clinical translation of LED-based PAI systems (more specifically, to obtain a detectable PA-signal from deep-seated tissue targets).

## 1. Introduction

Photoacoustic imaging (PAI) has been proven as a promising technology for nondestructive recovery of vital patho-physiological parameters (functional, structural, hemodynamics, mechanical, and molecular distribution [1,2,3,4,5,6] of biological tissues with a microscopic resolution at an unprecedented penetration depth (~cm) [7]. On the other hand, LED-based systems hold great potential in clinical translation because of their portability and affordability [8,9,10]. In LED-based PAI systems, the pulsed laser source is replaced by LEDs and, thus, the optical power of LEDs is insufficient to induce detectable photoacoustic (PA)-signal at higher penetration depths, which is the major drawback of the system [9]. Similar is true for the cases of laser diode based PAI systems [11]. In this context, enhancement of the achievable PA-signal strength is a longstanding problem in low energy light source based PAI systems and temperature dependent PA-signal enhancement is one of the most focusing areas of research studies in the past few years [12,13,14,15,16,17,18,19,20,21,22,23]. Enhancement in signal strength enables not only to improve obtainable imaging depth but also to increase accuracy in the quantitative measurement of physiological parameters by improving signal-to-noise-ratio (SNR) [12]. In this regard, an injection-based technique—in which exogenous contrast agents or target specific biomarkers are introduced to tissue sample of interest externally through injection—has been proven successful and remains the only standard technique [13,24,25]. Unfortunately, the technique suffers from serious drawbacks: (1) limited studies of patho-physiological activities due to limited availability or selection of target-specific biomarkers [24,25], (2) bio-compatibility, (3) bio-toxicity, and (4) invasive in nature because of the introduction of exogenous dyes—which are foreign substances—to body as well as breakage of the intervening tissues including skin. To our best knowledge, experimental studies on noninvasive techniques for enhancement of the PA-signal strength (or amplitude) are limited to the articles reported in Refs. [12,13,14,15,16,17,18,19,20,21,22,23]. In all of these reported studies for enhancement of PA-signal strength, imaging sample was immersed inside a heating bath filled with water that controls the thermodynamic equilibrium temperature (with an exception of the study [20] that controls the temperature of the imaging target by connecting an electrically conducting wire to imaging target of interest). Thus, all of the reported (experimental) techniques are not suitable for the end applications being targeted to clinical settings. Refs. [21,26,27] reported studies on theoretical aspects of the generation of PA-waves and its contribution from thermodynamic equilibrium temperature (*T*) and temperature raise (Δ*T*) induced by transient optical illumination. In our present article, we report a unique photothermal-based technique for enhancing the achievable PA-signal strength. Experimentally, enhancement is facilitated by pre-illumination of the sample with a continuous wave (CW)-optical beam (in addition to irradiation of the sample with short-pulse laser (with pulse-width ~ nsec)), thereby raising (thermodynamic equilibrium) temperature (*T*) of the imaging sample selectively over a pre-specified region of interest. Shortly, enhancement of PA-signal strength is achieved by control of thermodynamic property of imaging sample over a particular region of interest (unlike controlling temperature of entire sample or background as it is the case for the above mentioned or reported techniques). In this way, our proposed technique is noninvasive (without introducing contrast agents) and nondestructive (without damaging tissues).

With a close similarity to our proposed technique, in PAI-guided phototherapy or photothermal therapy [28,29,30], a CW-optical beam is employed to irradiate target-specific contrast dyes (being introduced to tissue targets), and the subsequent temperature raise (Δ*T*) is imaged by PAI modality. In the study [16], a CW-laser beam was used for inducing interstitial tissue coagulation while heating of the imaging targets was facilitated by hot air blow from a heat gun or conducting heating. Introduction of photo-absorbing contrast agents—like gold nanoparticles, nano-shells, and plasmonic nanoparticles—in the target region facilitates enhancement in contrast and photoacoustic signal [31,32,33]. This is an invasive procedure. On the other hand, recovery of the temperature distribution $\left(T(\vec{r})\right)$ of tissue samples with PA-imaging modality has been studied for more than a decade of years or so [12,14,15,16,23,34]. However, experimental techniques (including CW-irradiation) for enhancement of PA-signal strength from endogenous signal contrasts of (imaging) tissue sample and its clinical applications are not yet reported in literature. In this article, we propose a theoretical (analytical) hypothesis that derives a mathematical relationship of explicit dependence of PA-signal strength on ambient temperature (T) of the given physical system (tissue sample, in our case). This proposed theoretical hypothesis is validated with numerical simulation studies. For the simulation studies, we adopted k-wave (MATLAB) platform [35,36,37], which is widely employed as a standard numerical simulation tool for photoacoustic imaging. The experimental results, with experiments being performed in tissue-mimicking Agar phantom, as well as chicken breast, sample, demonstrate that the proposed photothermal-based technique can be adapted for clinical applications. In other words, the proposed technique was validated with experiments being conducted in tissue-mimicking phantom (Agar, in our case) as it is done conventionally in (biomedical) research laboratory settings [3,4,5,12,15,18] while the feasibility of clinical translation of the proposed technique was validated with experiments being conducted in tissues (chicken breast).

## 2. Materials and Methods

### 2.1. Theory

In PA-imaging, a beam of short laser pulses (pulse width ~ nsec) is delivered to the sample surface so as to irradiate a particular target of interest. Thermoelastic expansion occurs [38] due to rapid heating and subsequent cooling of the irradiated sample material. This results in the generation of pressure waves in the sample, which are known as initial photoacoustic pressure waves. One may consider the photoacoustic effect as a thermodynamic process [38]. The initial PA-pressure can be expressed as [3,38]:

(1) $$P_{0}=\;\frac{\beta }{\kappa }\frac{1}{{\rho c}_{V}}\mu_{a}\phi \;={\;\Gamma \mu }_{a}\phi .$$

where $\Gamma =\frac{\beta }{\kappa }\frac{1}{{\rho c}_{V}}$ is a dimensionless physical quantity, commonly referred as Grüeisen parameter, and it is a measure of thermoelastic efficiency of a given material. Here, in the case of PAI, *Γ* gives the measure of conversion efficiency from pulse optical energy to acoustic energy. We know that optical absorption coefficient ($\mu_{a}$) is characterized by optical extinction coefficient (ε) that can be expressed as:

(2) $$\mu_{a}=\;\varepsilon \left[C\right],$$

where $\left[C\right]$ is the concentration.

For a physical system or medium including solution of low concentration (methylene blue solution, as it is the case for our present study) and biological tissue, one can deduce Equation (1) as (a detailed derivation is provided in Appendix A):

(3) $$P_{0}=\;\frac{\beta^{\left(water\right)}}{\kappa^{\left(water\right)}}\;\frac{1}{\rho^{\left(water\right)}c_{V}^{\left(water\right)}}\varepsilon^{\left(methy\right)}{\left[C\right]}^{\left(methy\right)}\phi$$

Equation (3) implies that photoacoustic wave generation is dictated by the thermodynamic properties of the surrounding (background) medium/fluid (water, in our study). It is similar to the studies [21,22,23,26,27,39] that demonstrated PA-wave generation is dictated by thermodynamic properties of the fluid in which optical absorbing targets of vanishingly small size (point source or nanoparticles) were immersed. In view of the arguments (discussed in Appendix A), the above Equation (3) holds true for the case of Agar gel phantom where superscripts, (water) and (methylene blue) are replaced by (agar gel) and (ink), respectively. We adopt mathematical representation corresponding to methylene blue solution.

The reported study [40] gives the variation of thermodynamic parameters with (ambient) thermodynamic equilibrium temperature (*T*) (as it is given in Table 1). From Table 1, one can conclude that, in the temperature range (~ 20 °C to 40 °C), $\beta^{\left(water\right)}$ changes linearly with *T,* which can be represented by a straight line (*β^(water)^* = a + bΔ*T*, where ‘a’ and ‘b’ are intercept and slope, respectively). This assumption is in agreement with Taylor expansion of *β* close to equilibrium temperature $\left(T_{0}\right)$ given $by\;\beta \left(T_{0}\right)\to \beta \left(T_{0}+\Delta T\right)\approx \;\beta \left(T_{0}\right)+\Delta T\frac{d\beta }{dT}\;=\beta_{equil}+\Delta T\frac{d\beta }{dT},$ as it is done in Refs. [21,26,27], where Δ*T* is the differential change in temperature. $\beta_{equil}\;\left(\equiv \;\beta \left(T_{0}\right)\right)$ is the thermal expansion coefficient under thermodynamic equilibrium temperature $\left(T_{0}\right)$, which is again dependent on *T*. For water, thermal expansion coefficient (*β*) vanishes at *T* ~ 3.98 °C, i.e., *β*(*T*) = 0 for *T* ~3.98 °C [13,17]). At this zero-crossing temperature, from Equation (3), one can conclude that PA-waves cannot be generated under any circumstances (including increasing optical absorption coefficient (*µ_a_*) and/or optical fluence (*ϕ*)), which was validated by experimental studies in the past [21,26,41]. In our present study, we conducted experiments with T ~20–30 °C, which is far above the zero-crossing temperature (~4 °C) and the equilibrium term ($\beta_{equil}$) cannot be neglected [21]. In this temperature range of interest (~20–30 °C) for laboratory as well as clinical studies, from Table 1, we estimated ‘b’ to be ~9.64 °C and relative change of *β* is found to be ~46.63%. However, other thermodynamic parameters give negligible relative changes compared to *β*. Under these circumstances, we assume that $\kappa^{\left(water\right)}$, $\rho^{\left(water\right)}$, and $c_{V}^{\left(water\right)}$ are independent of T or constant in comparison to the dependence of $\beta^{\left(water\right)}$ on *T* [13,42]. Specific heat capacity ($c^{V}$) is independent of temperature (for soft tissue) over temperature range < 50 °C [16,43]. From Equation (3), we can obtain the explicit expression for *P*_0_ at an arbitrarily chosen temperature (T) in the neighborhood of thermodynamic equilibrium temperature (*T*_0_), i.e., *P*_0_ at $T_{0}\to T=T_{0}+\Delta T$ can be expressed as:

(4) $$P_{0}\left(T_{0}\right)\to \;P_{0}\left(T=T_{0}+\Delta T\right)=P_{0}\left(T_{0}\right)\;\frac{{\left[C\right]}^{\left(methy\right)}}{\alpha^{\left(water\right)}}\left((\varepsilon^{\left(methy\right)}\;{\left(\frac{\partial \beta^{\left(water\right)}}{\partial T}\right)}_{T_{0}}\Delta T\;+\;\beta^{water}\;{\left(\frac{\partial \varepsilon^{\left(methy\right)}}{\partial T}\right)}_{T_{0}}\Delta T\right)\phi$$

where $\alpha^{\left(water\right)}$ (=$\kappa^{\left(water\right)}\rho^{\left(water\right)}c_{V}^{\left(water\right)}$) and ${\left[C\right]}^{\left(methy\right)}$ are considered as independent of *T*. Equation (4) shows that the strength of initial PA-pressure waves (*P*_0_) is characterized by the ambient temperature $\left(T=T_{0}+\Delta T\right)$ of the medium in addition to $\mu_{a}$ and *ϕ*. In our present study, the differential raise in thermodynamic equilibrium temperature (Δ*T*) is facilitated by the pre-illumination of the sample with a CW-laser beam.

Equation (4) gives an explicit representation of *P*_0_ in integral form, into two separate thermal terms. The first term attributes to thermodynamic equilibrium temperature (*T*_0_), while the second term corresponds to transient changes in thermal expansion coefficient (*β*) and optical extinction coefficient (*ε*) that are resulted due to thermal perturbation in the physical (imaging) system by an external agency and its associated heating [44]. In our present study, we employed a CW-laser beam to raise the thermodynamic equilibrium temperature $T_{0}\to T_{0}+\Delta T$ and, thus, the second term in the generation of PA-signals (in Equation (4)) while pulse laser beam, which is contributing to the enhancement from the two terms, is kept fixed. Equation (4) shows that enhancement of strength of initial PA-pressure waves, being generated in mechanical medium (by transient optical illumination), can be achieved (experimentally) by control of ambient temperature (a) directly through heating of the imaging sample or by thermal technique and (b) indirectly through photo-illumination of the specimen raising incident optical power or photo-thermal technique. Direct method demands heating of the imaging specimen as a whole and, thus, it is not of much clinical importance (as it is discussed in Section 1). Indirect method facilitates to raise the temperature (*T*) of deep-seated tissue targets selectively over a pre-specified region of interest and, thus, this technique is of significant clinical impacts. Here, we focus on the indirect technique where we employed the CW-laser beam (for photo-illumination) in addition to the pulsed-laser beam that is adopted for transient illumination and subsequent generation of initial *P*_0_.

### 2.2. Enhancement of PA-Signal Strength

One can express relative enhancement in PA-signal strength, due to a raise in thermodynamic equilibrium temperature *T*_0_, as:

(5) $$P_{0}^{\left(enh\right)}\;=\;\frac{P_{0}\left(T=T_{0}+\;\Delta T\right)-P\left(T=T_{0}\right)}{P_{0}\left(T=T_{0}\right)}\times 100\;\left(in\;\%\right)$$

### 2.3. Numerical Simulation

**Figure 1:**
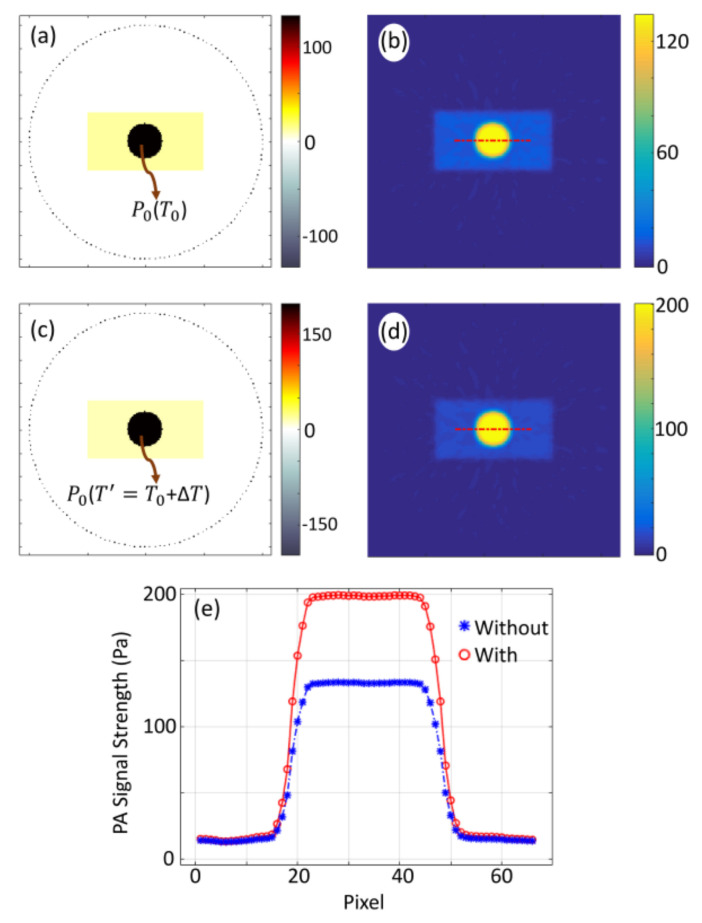
Representative images of initial pressure distribution of the tissue-mimicking numerical sample with a circular target being embedded in the background and sensor array distributed over a circular ring (**a**,**c**). Numerical samples with circular targets at a temperature $T_{0}$ (**a**), and temperature $T^{\prime }=T_{0}+\Delta T$ (**c**). The corresponding reconstructed images (**b**) for target at a temperature $T_{0}$ and (**d**) for target at temperature $T^{\prime }$ obtained using k-wave MATLAB toolbox. (**e**) Variation of PA-signal strength along the marked lines shown in (**b**,**d**).

A circular target of radius ∼3 mm having contrast in $\mu_{a}$ in comparison to that of the background is situated at the center of the background of area $21.6\;\times \;21.6{\;\mathrm{mm}}^{2}$. This numerical sample represents the deep-seated inhomogeneous target illuminated with CW-laser (to raise the temperature) in the background tissue medium. A circular sensor (diameter ∼20 mm) of 145 identical detectors is collecting the PA-waves for reconstruction as it is done in our previous study [45]. Figure 1a presents a representative image of the distribution of initial PA-pressure waves ($P_{0}$) at a temperature $T_{0}$ (say, room temperature), while Figure 1c presents the same at a temperature $T^{\prime }=\;T_{0}+\Delta T$ where ΔT is the temperature raise due to pre-illumination. The values of ΔT corresponding to different concentration $\left[C\right]$, absorption coefficient ($\mu_{a}$), and optical power are taken from experiment. Figure 1b,d present the reconstructed images corresponds to Figure 1a,c. Variation of PA-signal along the marked lines, which are indicated in Figure 1b,d, plotted in Figure 1e. We estimate the strength of initial PA-pressure waves, for the reconstructed pressure distribution, as the average value over full width at the 75% of the maximum of the line plots and it is found to be 130 Pa at room temperature $\left(T_{0}\right)$ and 193 Pa at a temperature $T^{\prime }=T_{0}+\Delta T$. (Here, ∆*T* is the temperature raise corresponding to a concentration of ∼7 mM). From these values, PA-signal enhancement due to temperature raise is calculated as 51%. In similar ways, we study variation PA-signal strength with change in temperature due to the changes in the physical parameters of the targets, and the results are plotted in Section 3.

### 2.4. Experimental Set-Up

**Figure 2:**
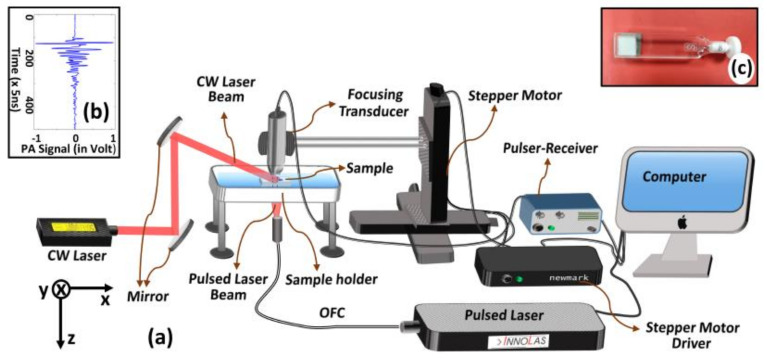
Schematic diagram of the experimental set-up (**a**). A typical 1D PA-signal acquired by our acoustic resolution photoacoustic microscopy (AR-PAM) imaging system (**b**). (**c**) Photograph of transparent cuvette (inner thickness ~1 mm) that is employed as holder for imaging sample (methylene blue solution).

For experiments, we employed a home-built acoustic resolution photoacoustic microscopy (AR-PAM) imaging system in transmission configuration. Figure 2a depicts a schematic diagram of the experimental set-up. A train of short duration pulses of optical beam—beam diameter ~2.3 mm, power ~25 mW, pulse width ~6 nsecs, pulse repetition frequency ~100 Hz, and wavelength ~670 nm—from a tunable pulsed OPO laser source (SpitLight OPO Evo 150–532, Innolas Lasers, Krailling, Germany) was coupled by an optical fibre (diameter ~6 mm) so as to irradiate tissue sample of interest. A tightly focusing ultrasound transducer (V375-SU, Olympus, Shinjuku City, Tokyo, Japan)—focal spot size ~154 µm (calculated using, $BD=1.02\;\frac{F*v}{f*D}$ [46], where *F* is focal length; *v* is speed of sound; *f* is operating frequency; *D* is the diameter of US transducer), operating frequency ~30 MHz, and focal length ~19.10 mm—was employed to pick-up transient light-induced PA-signals (*P*_0_) selectively from its narrow focal zone.

The detected PA-signals were amplified using a pulser-receiver (Part No.: 5073PR-40-P, Olympus, Shinjuku City, Tokyo, Japan) and, then, acquired using a data acquisition card (Part No.: 779745-02, NI PCI-5114, 250 MS/s, National Instruments, Austin, Texas, USA) being attached to a computer system. From time-resolved A-scan, depth-resolved data is obtained (as it was done in previous studies [3,5]). We obtained 2D or 3D data representative of PA-signals by raster scanning. We employed a high precession translation stage (Newmark NSC-G series, Newmark Systems Inc., Rancho Santa Margarita, CA, USA) for raster scanning of the transducer with step-size (~100 µm) while the optical pulse beam and the sample was kept fixed in a position. For a scanning area of ~4.8 × 3 mm^2^—that can be achieved by ~48 × 30 raster scanning steps—we can acquire an image at the frame rate ~0.69 Hz (~4 frames per min). In addition to pulse (nsec) laser beam, as it is shown in Figure 2a, the imaging sample is illuminated with a collimated optical beam (diameter ~3 mm) from a CW-laser source (Stradus-642-110, VORTRAN Laser Technology, Roseville, CA, USA; wavelength, 642 nm) in such a way that the CW-beam and pulse-laser beam intersect each other at a pre-specified region of interest. Optical wavelengths of CW-illumination (for enhancement of PA-signal strength) and PA-excitation (to induce PA-signals) can be selected independently. CW-laser is continuously switched on during the experiments to maintain the temperature. The entire AR-PAM imaging system is controlled by a LabVIEW-based software. Figure 2b gives a typical 1D PA-signal acquired by our AR-PAM imaging system, while Figure 2c depicts a photograph of the sample holder (transparent cuvette). During experiments, the cuvette filled with sample material (say, methylene blue solution) is completely immersed inside an acoustic-coupling medium (water, in our case) for proper coupling of acoustic signals. We employed AMIRA software for the generation of 3D images from a sequence of 2D images, which are, in turn, reconstructed by using MATLAB.

## 3. Experimental Results and Discussion

### 3.1. Enhancement of PA-Signal from Methylene Blue

**Figure 3:**
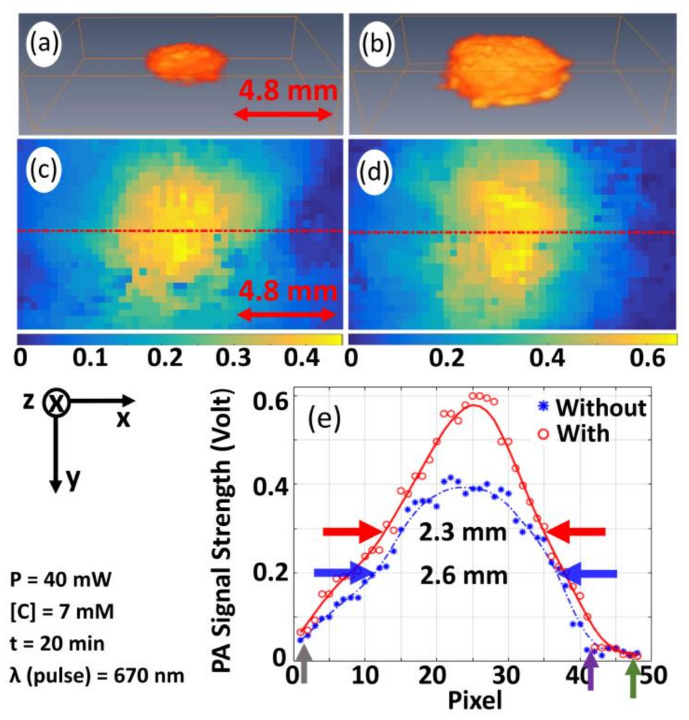
3D image representatives of $P_{0}$ without (**a**) and with (**b**) pre-illumination for methylene blue while 2D image corresponding to a randomly selected plane (at *z* = 7) ((**c**), without pre-illumination and (**d**), with pre-illumination). (**e**) Variation of $P_{0}$ along the marked lines depicted in (**c**,**d**). Arrowheads in both sides of scale bar (being included in (**a**,**c**)) indicate measure of the entire length of the image along *x*-axis. Arrowheads, marked in (**e**), indicate the points of observations for estimation of size of the illuminated spot.

Figure 3 gives the experimental results to compare PA-signal strengths ($P_{0}$) with and without pre-illumination of CW-laser beam. Experiments were conducted with methylene blue solution (concentration of ~7 mM using water as a solvent) as an imaging sample. Figure 3a,b present 3D image representatives of $P_{0}$ with and without pre-illumination, respectively, while Figure 3c,d give 2D images corresponding to a randomly selected plane (at z=7). For quantitative analysis, the variation of $P_{0}$ along the marked lines, which are indicated in Figure 3c,d, are plotted in Figure 3e. In Figure 3c,d, yellow spot appearing in blue background corresponds to $P_{0}$ acquired from pulse laser-irradiated spot in sample while the background corresponding to un-illuminated region, which gives no PA-signal. The size and shape of the image, which is characterized by pulse-laser spot, are in agreement with that of cross-section (FWHM) of pulse-laser beam. From Figure 3c,d, it is observed that maximum $P_{0}$ achievable with pre-illumination is 0.60 V against 0.40 V (without pre-illumination). Average $P_{0}$ over full-width at 75% of maximum is estimated to be 0.50 V (for pre-illumination) against 0.35 V (without pre-illumination) and enhancement ($P_{0}^{\left(enh\right)}$), corresponding to the plane z=7, is found to be 46%. In the similar fashion, $P_{0}^{\left(enh\right)}$ for the planes (corresponding to z=6 and z=8) were obtained as 40% and 38%, respectively. The average value of the measurements from the three consecutive planes $\left(41\%\right)$ is considered as the $P_{0}^{\left(enh\right)}$ with pre-illumination for a particular power and concentration. The results demonstrate that pre-illumination with CW-optical beam provides a significant enhancement in $P_{0},$ which is due to enhancement in temperature change following optical illumination of CW-beam (as it is given by Equation (5)). Similar experiments were conducted for empty cuvette and water (filled in cuvette) being employed as imaging sample. The experimental results, presented in supplementary figures (Figures S1 and S2), demonstrate that cuvette and water give no significant PA-signal for both of the two cases (with and without pre-illumination). These experimental results imply that $P_{0}^{\left(enh\right)}$ is not contributed from sample holder (cuvette) and acoustic-coupling medium (water), or enhancement is contributed only from the sample (methylene blue, in our case).

From Figure 3d, the size of the illuminated spot was estimated—for pre-illumination as well as without pre-illumination—from two different aspects: (a) measuring the full width at half maximum (FWHM) using four cross-sectional profiles (or line plots) (as it is shown in Figure S3). The profile along the *x*-axis is given in Figure 3e and FWHM corresponding to without (blue color arrow) and with (red color arrow) pre-illumination are estimated to be ~2.3 mm and ~2.6 mm, respectively. The average value of the measured FWHM from all profiles is calculated as ~2.3 mm (with) and ~2.2 mm (without), respectively. This shows that, by our proposed pre-illumination technique, the effective resolution is reduced. (b) Measuring the change in object size across the baseline, i.e., the distance between two points indicated in Figure 3e by arrowheads (grey color and violet color (without pre-illumination); grey color and green color (with pre-illumination)). The width of the spot was found to be ~4.3 mm (without pre-illumination) and ~4.7 mm (with pre-illumination) and the increment in width is obtained as 9.5%. This implies that the obtainable spatial resolution of the imaging system is slightly reduced $\left(\sim 9.5\%\right)$ by the proposed optical pre-illumination technique. This may be because of thermal excitation and diffusion of heat (resulted from pre-illumination of CW-laser beam) [18,22], thereby enhancing strength of PA-signal from the immediate background of the (pulse-laser) illuminated spot. More elaborately, transient heat generation, which results from transient pulse-laser excitation, is localized [47] (under the physical condition of thermal and stress confinement) while (ambient) temperature raise (and its heat generation) induced by CW-optical beam illumination is diffused (i.e., not localized over the illuminated region perfectly). In this way, the effective size of the illuminated spot becomes wider due to pre-illumination with CW-optical beam.

#### 3.1.1. Variation of PA-Signal Enhancement with Variation in Different Parameters

**Figure 4:**
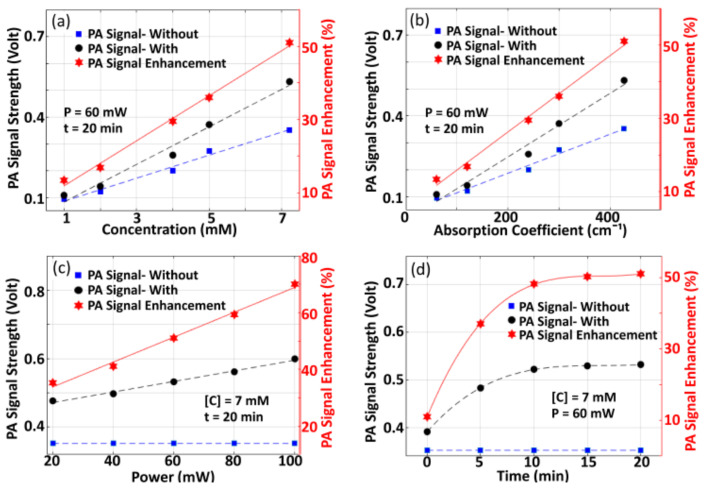
(**a**) Variation of measured $P_{0}$ and $P_{0}^{\left(enh\right)}$ with concentration $\left(\left[C\right]\right)$ of sample solution. In the experiments, we employed a continuous wave (CW)-laser of power $\left(P\right)\sim 60\;\mathrm{mW}$ and time duration of pre-illumination $\left(t\right)\sim 20\;\min$). (**b**) Variation of measured $P_{0}$ and $P_{0}^{\left(enh\right)}$ with optical absorption coefficient (where P=60 mW and t=20 min). (**c**) Variation of measured $P_{0}$ and $P_{0}^{\left(enh\right)}$ with optical power of CW-optical beam (where $\left[C\right]=7\;\mathrm{mM}$ and t=20 min), (**d**) Variation of $P_{0}$ and $P_{0}^{\left(enh\right)}$ with time-interval of pre-illumination (where $\left[C\right]=7\;\mathrm{mM}$, P=60 mW). In all of the experiments, we employed pulsed laser beam of wavelength, λ~670 nm.

Figure 4a presents the variation of $P_{0}$ with concentration $\left(\left[C\right]\right)$ of sample for both of the two cases (with pre-illumination and without pre-illumination) where $P_{0}$ is measured as an average over full-width at 75% of maximum as it is done in the previous case (shown in Figure 3e). In the experiments, we employed CW-laser with power $\left(P\right)\sim 60\;\mathrm{mW}$ and time duration of pre-illumination (*t*) ~20 min. Figure 4b depicts the variation of $P_{0}$ with respect to ${µ}_{a}$ (which is estimated from [C]), where P=60 mW and t=20 min being employed as the experimental parameters. Variation in $P_{0}^{\left(enh\right)}$ with pre-illumination, which is estimated using Equation (5), is also depicted. It is observed that $P_{0}$ and $P_{0}^{\left(enh\right)}$ increases linearly with concentration. The observed linearity may be because of an increase in deposition of optical energy as well as mechanical coupling of constituent particles with an increase in $\left[C\right]$. Variation in $P_{0}$ and $P_{0}^{\left(enh\right)}$ with CW-optical power used for pre-illumination is shown in Figure 4c, (where $\left[C\right]=7\;\mathrm{mM}$ and t=20 min being employed as the experimental parameters), while $P_{0}^{\left(enh\right)}$ with time interval (*t*) of pre-illumination of CW-optical beam is depicted in Figure 4d (where $\left[C\right]=7\;\mathrm{mM},\;P=60\;\mathrm{mW}$). Without pre-illumination, the optical power of incident pulse-beam remains the same so that $P_{0}$ remains unchanged (which are depicted by blue-color dotted lines in Figure 4c,d). In this case, $P_{0}$ and $P_{0}^{\left(enh\right)}$ increases non-linearly, which is followed by saturation at a particular time-point t~20 min. This is due to saturation in the deposition of optical energy and, thus, thermo-elastic expansion. Saturation in $P_{0}^{\left(enh\right)}$ suggests that, for practical applications, selection of stability point of signal enhancement is demanded. In our validation study, with experiments being conducted in tissue-mimicking Agar phantom and chicken tissue, pre-illumination of the sample with t=20 min was performed.

#### 3.1.2. Variation of Temperature Raise with Variation in Different Parameters

**Figure 5:**
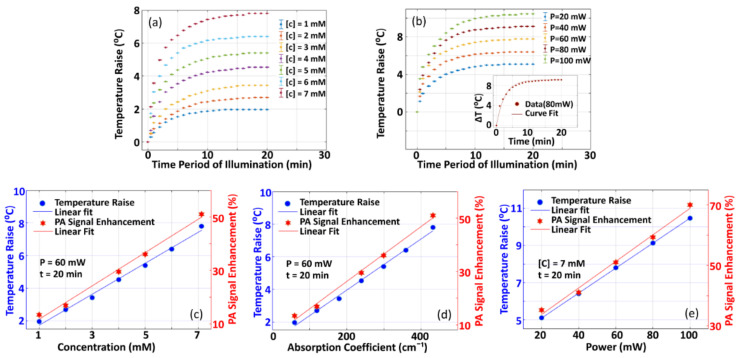
(**a**) Variation of temperature-raise $\left(\Delta T\right)$ with respect to time-interval $\left(t\right)$ of pre-illumination of CW optical beam (with concentration as experimental parameter (for P=60 mW)). (**b**) Variation of ΔT against t with optical power $\left(P\right)$ of CW laser beam as an experimental parameter (for $\left[C\right]=7\;\mathrm{mM}$), (**c**) Variation of ΔT against concentration (where P=60 mW, t=20 min were employed), (**d**) Variation of ΔT against optical absorption coefficient (where P=60 mW and t=20 min were employed), and (**e**) Variation of ΔT against optical power of CW beam for pre-illumination (where $\left[C\right]=60\mathrm{mW}$ and t=20 min were employed).

Figure 5 presents the experimental results for characterization studies of the variation of temperature-rise (ΔT) with various physical parameters of interest. A detailed description of the experimental set-up employed for this study is provided in supplementary (see Figure S4). Figure 5a depicts the variation of ΔT with respect to the time interval of pre-illumination $\left(t\right)$ of the CW-optical beam where concentration $\left(\left[C\right]\right)$ of methylene blue is considered as an experimental parameter. Meanwhile, the variation of ΔT with t, where optical power of CW-optical beam is considered as an experimental parameter, is shown in Figure 5b. In these figures, it is observed that the temperature raise increases non-linearly and attains saturation at a certain point of the time interval of pre-illumination (~20 min). Inset in Figure 5b shows a curve-fit, for 80 mW power of CW-laser, using $\Delta T=\;\Delta T_{sat}\left(1-\;e^{-\gamma t}\right)$ (where $\Delta T_{sat}$ is the maximum temperature raise attained at saturation and γ is a constant that characterizes the growth rate) and it is found that maximum temperature raise is 9.13 °C while growth rate, $\gamma \;\sim \;0.3909{\;\sec}^{-1}$. From the figures, it is observed $\Delta T_{max}$ and γ are dependent on $\left[C\right]$ and optical power of incident CW-beam. Figure 5c shows the variation of temperature raise with $\left[C\right],$ which are obtained from Figure 5a with t=20 min. From Figure 5c, we obtained variation of ΔT with ${µ}_{a}$ and it is shown in Figure 5d. Similarly, the variation of ΔT with optical power of CW-beam at t=20 min is given in Figure 5e. In the figures (Figure 5a–e), $P_{0}^{\left(enh\right)}$, which is presented in Figure 4, is also presented for comparison of ΔT and $P_{0}^{\left(enh\right)}$. This linearity in ΔT—which is due to increase and subsequent saturation in the absorption of optical energy—is in agreement with that for $P_{0}^{\left(enh\right)}$. From these results (Figure 4 and Figure 5), one may conclude that $P_{0}^{\left(enh\right)}$ is dominantly contributed from ΔT due to CW-optical pre-illumination in support of our theoretical hypothesis given by Equation (4). This is in agreement with the (simulation and experimental) study, reported by Mahmood et al. [20], that demonstrated Γ and, thus, $P_{0}$ are dependent on temperature. The time duration for achieving the saturation point of signal enhancement is characteristic of samples, and it can be considered as a preparatory requirement for imaging.

### 3.2. Variation of Optical Extinction Coefficient of Methylene Blue with Temperature

**Figure 6:**
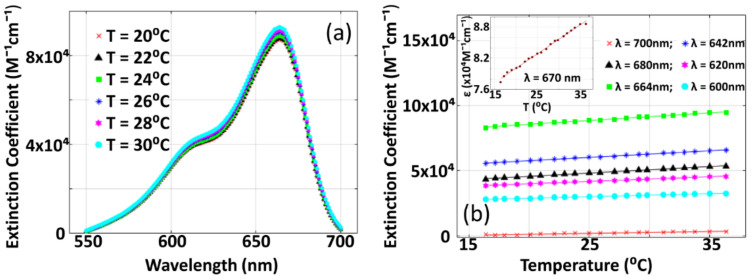
Variation of extinction coefficient (**a**) with respect to optical wavelength (λ) (at various thermodynamic temperature (T)). (**b**) Variation of optical extinction coefficient (ε) against (T) for different λ. Inset in (**b**) presents variation of ε against T for λ=670 nm, which is the optical wavelength of pulsed laser beam used for imaging (in our present study). In the experiments, we used methylene blue solution with $\left[C\right]∼2\;\mu M$.

**Figure 7:**
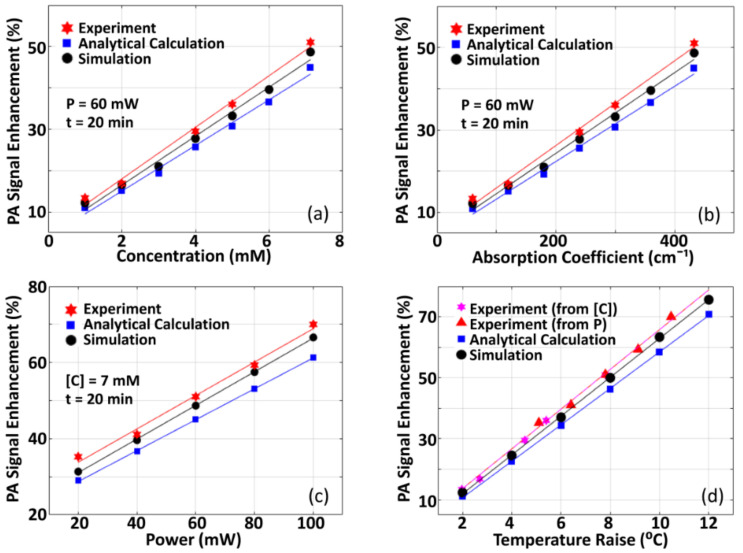
Comparison of the results obtained from experiments, theoretical hypothesis, and numerical simulation. (**a**) Variation of $P_{0}^{\left(enh\right)}$ with concentration (we employed P=60 mW and t=20 min), (**b**) Variation of $P_{0}^{\left(enh\right)}$ with optical absorption coefficient (P=60 mW and t=20 min were employed). (**c**) Variation of $P_{0}^{\left(enh\right)}$ with optical power ($where\;\left[C\right]=7\mathrm{mM}\;and\;t=20\;\min$). (**d**) Experimental measurements in $P_{0}^{\left(enh\right)}$ with respect to temperature raise (ΔT), with ΔT being obtained from variation of optical power as well as concentration. The wavelength of the pulsed laser used for experiment is 670 nm.

Figure 6 gives the experimental results for the characterization study of variation of optical extinction coefficient (ε) with temperature. For this study, we employed a UV-VIS as spectrum analyzer (PerkinElmer, Lambda 750) and ε is estimated from absorbance spectrum using $\varepsilon =\frac{Absorbance}{d\;\left[C\right]}$ where d is the (inner) thickness of sample holding cuvette $\left(\sim 1\;\mathrm{cm}\right)$ where methylene blue solution is filled in. The sample is heated before introduction to the UV-VIS spectrum analyzer. Temperature (T) is measured before and after the experiments, and its mean value is considered as the temperature measurement for the particular spectrum analysis experiment. Figure 6a depicts the variation of *ε* against wavelength $\left(\lambda \right)$ while Figure 6b gives the variation of ε against T. From the figure, it is observed that *ε* varies linearly with T where intercept and slope of the linear curve are characteristics of λ. From this experimentally estimated *ε*, for a given T, we estimated the contribution of optical absorption to the enhancement of photoacoustic signal strength (given in Equations (4) and (5)). However, thermal expansion coefficient at any given temperature T was estimated using a linear fit of β with T given in Table 1. Using these measurements of ε and β (in Equations (4) and (5)), we estimated $P_{0}^{\left(enh\right)}$, which we consider as analytical calculation (in Figure 7), wherein thermal perturbation (ΔT) is adopted from the measurements corresponding to Figure 5.

### 3.3. Comparison of Numerical Simulation and Experimental Results, and Validation of Theoretical Hypothesis

Figure 7 presents the results of numerical simulation studies—for validation of our proposed theoretical hypothesis (presented in Section 2.1)—in comparison to the experimental results (given in Figure 4). In the meantime, $P_{0}^{\left(enh\right)}$, which is estimated theoretically or analytically using Equations (4) and (5), is also included. Variation of $P_{0}^{\left(enh\right)}$ with respect to [C], ${µ}_{a}$, optical power of CW-beam (used for pre-illumination), and ΔT are shown in Figure 7a–d. We estimate the slopes for variation of $P_{0}^{\left(enh\right)}$ with: (i) concentration as 6.24/mM (from experiments) against 5.52/mM (from analytical calculation) and 5.92/mM (from simulations), (ii) optical absorption coefficient as 0.103 cm (from experiments) against 0.091 cm (from analytical calculation) and 0.098 cm (from simulations), (iii) optical power of CW-beam as 0.439/mW (from experiments) against 0.406/mW (from analytical calculation) and 0.442/mW (from simulations), (iv) temperature raise (due to concentration or optical power) as $5.97\;{}^{\circ}C^{-1}\;$(from analytical calculation) and $6.37\;{}^{\circ}C^{-1}$ (from simulations). In the figures, it is observed that slopes of the variation of $P_{0}^{\left(enh\right)}$ with the physical parameters of interest, obtained from experiments and numerical simulations, are in good agreement. This demonstrates that the proposed hypothesis of the dependence of $P_{0}$ and $P_{0}^{\left(enh\right)}$ on T (as it is given in Equations (4) and (5)) is validated. A deviation in absolute measurements of enhancement, as it is observed in Figure 7, may be due to lower in measurement of temperature of the imaging sample at a pre-specified region of interest—over which CW laser beam illuminates—resulting from tips of the (thermocouple) temperature sensor not being situated exactly at the point of illumination while attempting to prevent direct exposure of the tips to the incident light beam. To note, direct exposure of light to thermocouple tips results in the heating of the tips, which gives inaccurate measurements of the temperature of the medium.

### 3.4. Validation of the Proposed Technique for Pre-Clinical/Clinical Studies

**Figure 8:**
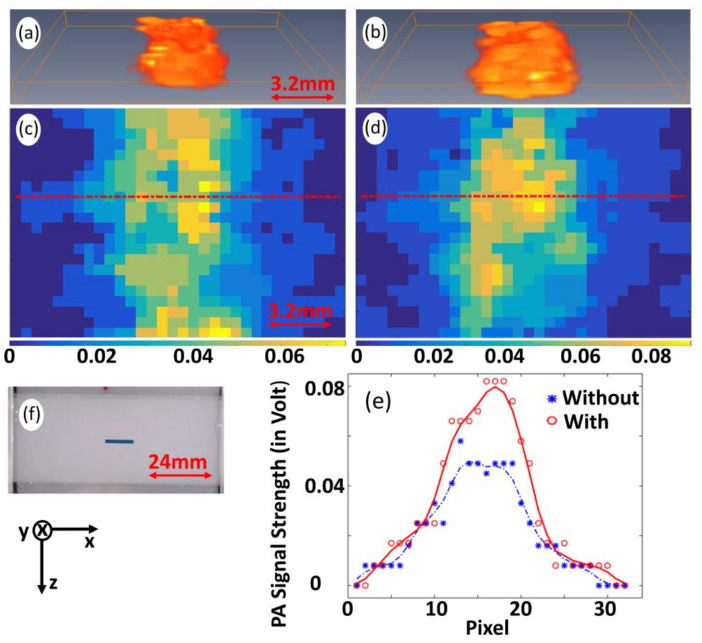
3D images representative of $P_{0}$ obtained without (**a**) and with pre-illumination (**b**), while (**c**,**d**) give corresponding 2D images for plane (at y=3). (**e**) Variation of $P_{0}$ and $P_{0}^{\left(enh\right)}$ along marked lines indicated in (**c**,**d**). Photograph of tissue-mimicking Agar-sample (**f**) where marked line indicates region for raster scanning. Arrowheads in both sides of the scale bar (being included in the figure) indicate measure of entire length of the image along x -axis.

**Figure 9:**
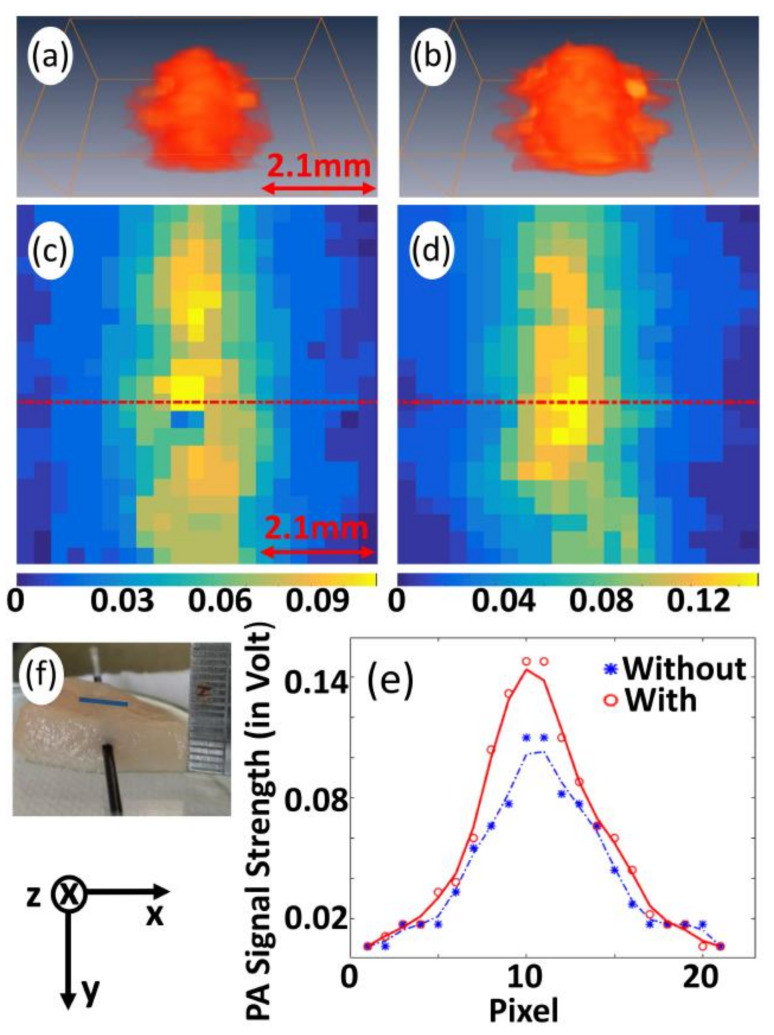
3D images representative of $P_{0}$ obtained without (**a**) and with pre-illumination (**b**), while (**c**,**d**) give corresponding 2D images for plane (at y=3). (**e**) Variation of $P_{0}$ and $P_{0}^{\left(enh\right)}$ along marked lines indicated in (**c**,**d**). Photograph of chicken breast tissue (**f**) in which transparent glass of inner diameter (∼1 mm) filled with methylene blue, thereby, mimicking deep-seated blood vessels is inserted at depth ∼2.5 mm (as it is observed from attached scale). Marked line indicates region for raster scanning. Arrow heads in both sides of scale bar (being included in the figure) indicate measure of entire length of the image along x -axis.

To validate the proposed technique for enhancement of PA-signal strength, in preclinical and/or clinical studies, experiments were conducted in tissue-mimicking Agar-phantom as well as biological tissue (chicken breast collected from supermarket). Experimental results are depicted in Figure 8 (for Agar phantom) and Figure 9 (for chicken tissue). Figure 8a,b give 3D images representative of $P_{0}$ obtained without and with pre-illumination. Figure 8c,d depict 2D images (corresponding to y=3 in 3D images). Line plots, showing the variation of $P_{0}$ and $P_{0}^{\left(enh\right)}$ along marked lines indicated in Figure 8b,c, are depicted in Figure 8d. $P_{0}^{\left(enh\right)}$ is found to be 48%, which is in agreement with $∼1.5\%\;{}^{\circ}C^{-1}$ increase in the Grüneisen parameter with temperature (for biological tissue) [8,20]. It is observed that the measured $P_{0}$ is significantly enhanced with pre-illumination of CW-laser beam. Figure 8f gives a photograph of Agar-sample where a target ($1.2\times 0.5\times 2.2\;{\mathrm{mm}}^{3}\left(xyz\right)$) was embedded in a background phantom ($24\times 1.5\times 10\;{\mathrm{mm}}^{3}$). Similar to reported studies [3,5], optical and elastic properties were tailored such that the target has only contrast in $\mu_{a}$ with respect to background ($0.02\;{\mathrm{mm}}^{-1}$ (target) against $0.01{\;\mathrm{mm}}^{-1}$ (background)). Figure 9a,b gives 3D images, while Figure 9c,d give 2D images, representative of $P_{0}$ obtained without and with pre-illumination. Line plots, showing the variation of $P_{0}$ and $P_{0}^{\left(enh\right)}$ along the marked lines indicated in Figure 9b,c, are depicted in Figure 9d. $P_{0}^{\left(enh\right)}$ is estimated and found to be ∼40%, which is a significant enhancement with pre-illumination of CW-laser beam. Figure 9f gives a photograph of the chicken breast tissue sample where a transparent (glass) tube of inner diameter (∼1 mm) is inserted through the chicken breast sample at depth ∼2.5 mm. The tube was filled with methylene blue, whereby mimicking deep-seated blood vessels in (chicken breast) tissue sample. In Figure 8, one observes that PA-representative image of the rectangular-shaped target appears to be circular (against appearing as a line in Figure 8c and Figure 9c). This is because of the pre-illumination of CW-laser beam, which is circular in cross-section. These results, with experiments being conducted in (chicken) tissue sample, demonstrate the practical applicability of the proposed photo-thermal technique to pre-clinical and/or clinical studies.

### 3.5. Discussion

The enhancement of PA-signal strength can be contributed from various factors (including change in specific heat capacity and isothermal compressibility), which are neglected in view of the argument described in Section 2.1. In our study, we assume that signal strength enhancement is contributed from a raise in thermodynamic equilibrium temperature $\left(T\right)$ and, thus, thermal expansion coefficient (β) and optical extinction coefficient (ε) of the tissue target. Moreover, the CW-laser beam enhances (but does not induce itself) PA-signal strength induced by pulse-laser excitation. Even though the CW-laser illumination increases the optical energy being irradiated to sample, the total optical energy is restricted within this safety limit $(∼20\;\mathrm{mJ}/{\mathrm{cm}}^{2}$, FDA, Silver Spring, MD, USA [48]). Experiments were carried out to compare efficiency in the enhancement of PA-signal strength with CW-laser pre-illumination and pulse-laser excitation. Experimental results demonstrate that it is more efficient (46% (for CW-laser pre-illumination) against 98% (for pulsed-laser excitation)) to increase PA-signal strength with an increase of pulsed-laser energy in comparison to a similar amount of increase in CW-laser energy, which is in agreement with the reported study [27]. Even though pulse-laser is more efficient to enhance PA-signal strength, the price of pulse-laser increases drastically as the output pulse energy increase, which then leads to an increase in the overall cost of the PAI system. In this way, the proposed technique paves a cost-effective way to improve PA-signal strengths. Again, from our study, it is observed that a temperature raise due to illumination of CW-optical beam is ∼10.5 °C, which is very low in comparison to temperature increase as in the case of photo-therapy (where targeted temperature measures ∼40 °C [28,43,49]), which remains as a standard clinical therapy. To the best of our knowledge, this pre-illumination technique may not impose any significant side effects in tissue (in in-vivo study and its applications).

Refs. [14,15] demonstrated that with an increase in temperature ∼37 °C (from ∼33 °C to ∼70 °C), PA- signal strength is enhanced by ∼120% (from ∼5 mV to ∼11 mV) for soft tissue (chicken breast) and ∼116% (from ∼6 mV to ∼13 mV) (for porcine liver) [14,15]. For porcine muscle, enhancement in PA-signal strength is ∼42% corresponding to change in temperature ∼9 °C [28]. The enhancement of PA-signal strength we achieved in our study is relatively high in comparison to that of Refs. [14,15,16]. This may be because of different experimental conditions. In our study, a deep-seated target of interest is selectively illuminated by CW-laser beam and, thus, the target is selectively heated while in Ref. [14], the entire sample is immersed inside a heating bath, and the temperature is raised as a whole including background (that also generate PA-waves), i.e., the difference in the enhancement of PA-signal strength may be due to reduce in the obtainable SNR resulted from the increasing background signal [12]. In Ref. [21], an optical fluence ∼$5\;\mathrm{mJ}/{\mathrm{cm}}^{2}$ was employed against ∼$15\;\mathrm{mJ}/{\mathrm{cm}}^{2}$ in our present study that gives a difference of 6 times in enhancement of signal strength.

One of the drawbacks of our proposed photo-thermal technique is that enhancement of PA-signal strength is at the cost of degradation in the obtainable spatial resolution. However, the degradation in spatial resolution is ~9%, which is much lower than the enhancement in PA-signal strength (~70% (methylene blue), ∼48% (Agar phantom)), and ~40% (chicken tissue)). Our study suggests that one can employ a CW-optical beam to perform pre-illumination specifically over a certain point deep inside the tissue without disturbing the intervening tissue medium so as to further enhance the PA-signal strength from a specific deep-seated target reducing the noise from the background or surroundings. LED-based PAI is recently gaining popularity in a wide range of superficial imaging applications, and it holds greater potential in clinical translation because of its portability and affordability. The proposed technique is a promising method to image deep-seated tissues with LED-based systems and accelerates its clinical translation. Strengthening the intensity of boundary measured signals—for improving signal detectability—is a critical factor in imaging (in general) and PA-imaging (in particular). In addition, the obtainable signal contrast between targets (including contrast agents) and the background is more relevant to the improvement of imaging quality. Our proposed photo-thermal technique offers improvement in signal contrast and, thus, the image quality.

## 4. Conclusions

In conclusion, we demonstrate an optothermal-based technique for enhancement of obtainable PA-signal strength that is, experimentally, facilitated by pre-illumination with CW-optical beam (in addition to pulse-optical beam) in imaging sample. We propose a theoretical hypothesis and it is validated by numerical simulation studies being performed using k-wave toolbox. Experimental studies were conducted in tissue-mimicking Agar phantom and ex-vivo animal tissue (chicken breast) samples. Experimental results demonstrate that a significant enhancement (up to ~70% (methylene blue), ~48% (Agar phantom), and ~40% (chicken tissue)) in the measured PA-signal strength can be achieved by our proposed photo-thermal technique. This unique (non-invasive and non-destructive) technique for enhancement of PA-signal strength will have a significant impact in PA-imaging while improving not only achievable penetration depth but also SNR and, hence, accuracy in quantitative measurement of vital patho-physiological parameters. Particularly, this technique can address the pertaining challenge associated with weak PA-signal from deep-seated tissue regions, which is the major issue associated with the clinical translation of the LED-based PAI systems.

## Figures and Tables

**Table 1:** Dependence of thermodynamic parameters on thermodynamic equilibrium temperature (*T*) for water.

Temperature (°C or K)	*β^(water)^* (10^−6^ K^−1^)	*κ^(water)^* (10^−10^ Pa^−1^)	*⍴^(water)^* (kg m^−3^)	*C_P_^(water)^* (kJ kg K^−1^)	*C_V_^(water)^* (kJ kg K^−1^)
20 or 293	206.80	4.5891	998.21	4.1818	4.1545
30 or 303	303.23	4.4770	995.65	4.1784	4.1159
40 or 113	385.30	4.4240	992.22	4.1785	4.0826

## Data Availability

The data that supports the findings of the study are provided within the article and the supplementary material.

## Supplementary Materials

The following are available online at https://www.mdpi.com/1424-8220/21/4/1190/s1, Figure S1: 2D images representative, for empty cuvette as sample specimen for imaging, of PA-signal strength obtained without and with pre-illumination, Figure S2: 2D images representative, for water (filled in cuvette) as sample specimen for imaging, of PA-signal strength obtained without and with pre- illumination, Figure S3: 2D images (corresponding to Fig. 3) and line plots along different direction for the calculation of FWHM, Figure S4: A schematic diagram of experimental set-up for the study of variation of temperature rise.

## Appendix A. Dependence of Initial Pressure Waves on Thermodynamic Parameters for Low Concentration Materials

For a physical system or medium (including solution and biological tissue), that is constituted by various individual components, its mass density is given by *ρ* $\approx \frac{\sum_{i=1}^{N}m_{i}}{\sum_{i=1}^{N}V_{i}}$ (where $m_{i}$ and $V_{i}$ are mass and volume of $i^{th}$ component, and *N* is the total number of individual components). Under physical condition of low concentration, as it is the case of our present study (methylene blue solution with concentration of methylene (∼mM) being dissolved in water (as a solvent) and tissue-mimicking Agar phantom with concentration of India ink (∼ 3–5 μl) in Agar gel (∼ 100 gms) [3,4,5]), one can approximate $\sum_{i=1}^{N}m_{i}\;\approx m^{\left(water\right)}$ and $\sum_{i=1}^{N}V_{i}\;\approx V^{\left(water\right)}$ (for methylene blue solution), i.e., $\rho \approx \rho^{\left(water\right)}$ (for methylene blue solution) and $\rho \;\approx \;\rho^{\left(agar\right)}$ (for tissue-mimicking Agar phantom added with India ink as an optical absorber). Similarly, specific heat capacity can also be approximated as $c_{V}=\;\frac{\sum_{i=1}^{N}Q_{i}}{\sum_{i=1}^{N}m_{i}}$ $\approx \frac{Q^{\left(water\right)}}{m^{water}\Delta T}=c_{V}^{\left(water\right)}$ (where $Q_{i}$ is the heat absorbed by $i^{th}$ components of the medium). In the meantime, optical properties of the system is given by collective effects of the individual components as linear combination [50], i.e., $\mu_{a}=\;\sum_{i=1}^{N}\mu_{a}^{i}\;=\;\sum_{i=1}^{N}\varepsilon^{i}\left[C^{i}\right]$ (where $\varepsilon^{i}$ and $\left[C^{i}\right]$ are extinction (optical absorption) coefficient and concentration of ith component, respectively). For methylene blue solution, we can approximate that $\mu_{a}=\;\mu_{a}^{\left(water\right)}+\;\mu_{a}^{\left(methy\right)}$ = $\mu_{a}^{\left(water\right)}+\;\varepsilon^{\left(methy\right)}[C^{\left(methy\right)}]\;\approx \;\mu_{a}^{\left(methy\right)}$ (for $\mu_{a}^{\left(water\right)}\ll \;\mu_{a}^{\left(methy\right)}$ [37]). Similarly for Agar phantom, one can approximate $\mu_{a}=\;\mu_{a}^{\left(agar\right)}+\;\mu_{a}^{\left(ink\right)}$ = $\varepsilon^{\left(agar\right)}[C^{\left(agar\right)}]+\;\varepsilon^{\left(ink\right)}[C^{\left(ink\right)}]\;\approx \;\varepsilon^{\left(ink\right)}[C^{\left(ink\right)}]$ $\approx \;\mu_{a}^{\left(ink\right)}$ (for $\varepsilon^{\left(agar\right)}\;\ll \;\varepsilon^{\left(ink\right)}$ [51]). Again, from Dalton’s law of partial pressures, total pressure of a thermodynamic system is given by an algebraic sum of partial pressures imparted by the individual components, i.e., $P=\;\sum_{i=1}^{N}P_{i}$ (where $P_{i}$ is the partial pressure of $i^{th}$ component) and it can be approximated as $P\approx P^{\left(water\right)}$ (for low concentration methylene solution) or $P\approx P^{\left(agar\right)}$ (for Agar phantom). Further, under thermodynamic equilibrium condition, there exists a uniform distribution of temperature $\left(T\right)$ at any arbitrarily chosen (spatial) point $\left(\vec{r}\right)$ over the entire space of the thermodynamic system, i.e., T is independent of spatial position $\left(\vec{r}\right)$ and, thus, measurements of T for all of the individual constituent components are the same. From the thermodynamic relations of κ and β, we can approximate $\kappa =\;-\frac{1}{V}{\left(\frac{\partial V}{\partial P}\right)}_{T}\approx \;-\;\frac{1}{V^{\left(water\right)}}{\left(\frac{\partial V^{\left(water\right)}}{\partial P^{\left(water\right)}}\right)}_{T}=\;\kappa^{\left(water\right)}$ and $\beta =\;-\frac{1}{V}{\left(\frac{\partial V}{\partial T}\right)}_{P}\approx -\;\frac{1}{V^{\left(water\right)}}{\left(\frac{\partial V^{\left(water\right)}}{\partial T^{\left(water\right)}}\right)}_{P}=\;\beta^{\left(water\right)}$ (for methylene blue solution) while $\kappa =\;-\frac{1}{V}{\left(\frac{\partial V}{\partial P}\right)}_{T}\approx \;-\;\frac{1}{V^{\left(water\right)}}{\left(\frac{\partial V^{\left(agar\right)}}{\partial P^{\left(agar\right)}}\right)}_{T}=\;\kappa^{\left(agar\right)}$ and $\beta =\;-\frac{1}{V}{\left(\frac{\partial V}{\partial T}\right)}_{P}\approx \;-\;\frac{1}{V^{\left(agar\right)}}{\left(\frac{\partial V^{\left(agar\right)}}{\partial T^{\left(agar\right)}}\right)}_{P}=\;\beta^{\left(agar\right)}$ (for Agar phantom). Now, for the case of low concentration, we can write Equation (A1) as:

(A1) $$P_{0}=\;\frac{\beta^{\left(water\right)}}{\kappa^{\left(water\right)}}\frac{1}{\rho^{\left(water\right)}c_{V}^{\left(water\right)}}\mu_{a}^{\left(methy\right)}\phi$$
