# Supplementary material for: Enhancement of Photoacoustic Signal Strength with Continuous Wave Optical Pre-Illumination: A Non-Invasive Technique
Source: Sensors (Basel). 2021 Feb 8;21(4):1190. doi: 10.3390/s21041190 (PMC7914629; doi:10.3390/s21041190)
Supplement: Supplementary file 1 [file sensors-21-01190-s001.pdf]

sensors

Article

Enhancement of Photoacoustic Signal Strength with Continuous Wave Optical Pre-Illumination: A Non-Invasive Technique

Anjali Thomas [1], Souradip Paul [1], Joy Mitra [2] and Mayanglambam Suheshkumar Singh [1,*]

[1] Biomedical Instrumentation and Imaging Laboratory (BIIL), School of Physics (SoP), Indian Institute of Science Education and Research Thiruvananthapuram (IISER-TVM), Thiruvananthapuram 695551, India; anjalithomas16@iisertvm.ac.in (A.T.); souradip.rkm16@iisertvm.ac.in (S.P.)
[2] Scanning Probe Microscopy and Plasmonics Lab, School of Physics (SoP), Indian Institute of Science Education and Research Thiruvananthapuram (IISER-TVM), Thiruvananthapuram 695551, India; j.mitra@iisertvm.ac.in
* Correspondence: suhesh.kumar@iisertvm.ac.in

Supplementary Materials:

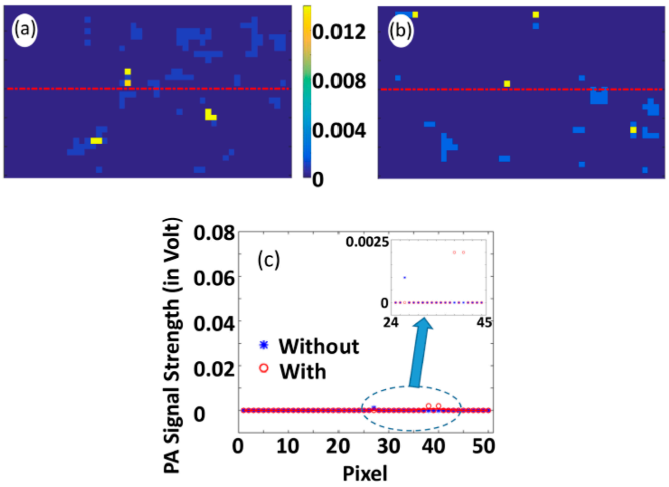

Figure S1. 2D images representative, for empty cuvette as sample specimen for imaging, of PA-signal strength obtained without (**a**) and with pre-illumination (**b**) (3D images are not presented here). (**c**) Variation of PA-signal strength and its enhancement along marked lines indicated in (**a**) and (**b**). It is observed that water gives no detectable PA-signal strength (with an exception of background noise that gives PA-signal strength ($\sim mV$ after amplification with a factor $39dB \equiv 90$).

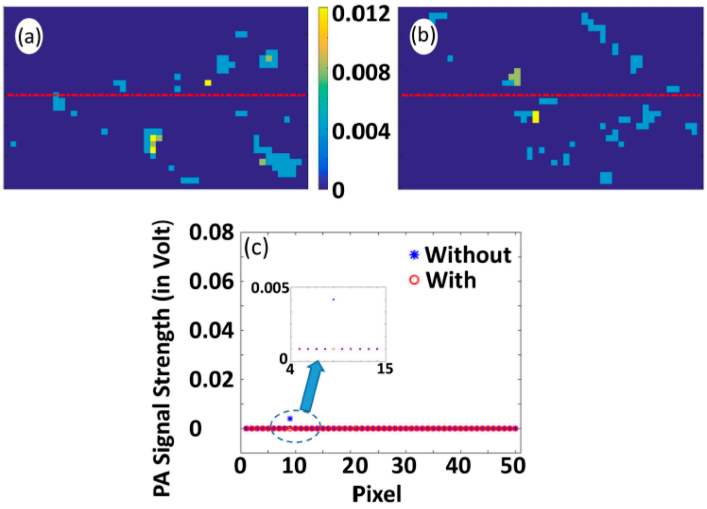

Figure S2. 2D images representative, for water (filled in cuvette) as sample specimen for imaging, of PA-signal strength obtained without (**a**) and with pre- illumination (**b**) (3D images are not presented here). (**c**) Variation of PA-signal strength and its enhancement along marked lines indicated in (**a**) and (**b**). It is observed that water gives no detectable PA-signal strength (with an exception of background noise that gives PA-signal strength ($\sim mV$ after amplification with a factor $39dB \equiv 90$).

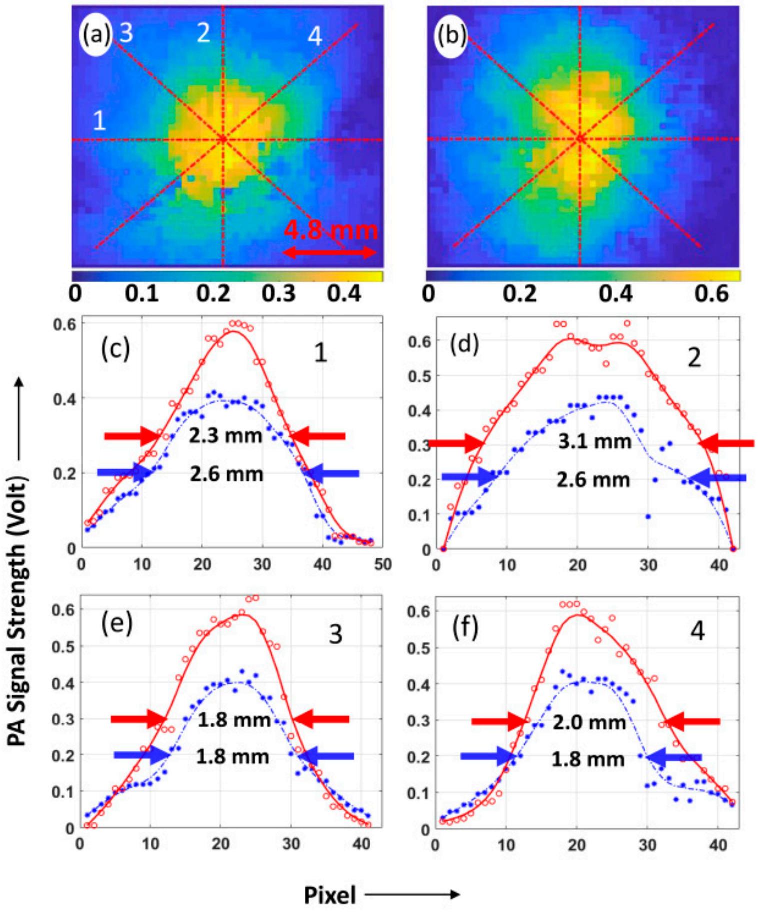

Figure S3. 2D images (corresponding to Fig. 3) and line plots along different direction for the calculation of FWHM. (**a**) Without pre-illumination and (**b**) with pre-illumination. (**c**) Profile (line plot) along line 1 marked in (**a**), (**d**) along line 2, (**e**) along line 3 and (**f**) along line 4. Red marking corresponding to with pre-illumination and blue corresponding to without pre-illumination. The average value of the measured FWHM from all profiles is calculated as $\sim 2.3mm$ (with) and

~2.2mm (without) respectively. From these values, one can conclude that the resolution is degrading slightly with pre-illumination.

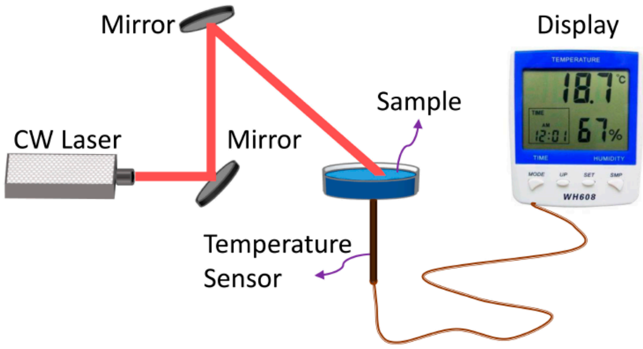

Figure S4. A schematic diagram of experimental set-up for study of variation of temperature rise (ΔT). The sample (methylene blue) is taken in a petri dish and is illuminated with the 642nm CW laser (Stradus 642nm, VORTRAN Laser Technology; wavelength, 642nm) such that the laser beam directly falls on the sample. In order to measure the temperature variation, a thermocouple temperature sensor (SS110, PT100 Sensor, Daihan Scientific, Jijeong-myeon, South Korea) is inserted into the sample, which is then connected to a digital display unit (WiseStir SMHS- 6, Daihan Scientific, Jijeong-myeon, South Korea) from where the values are noted. As it is shown in the figure, the thermocouple and the optical beam are aligned in a particular con- figuration that the optical beam does not incident directly on the thermocouple tips. Otherwise, instead of giving reading and measurement of temperature of the immersing solution, the thermocouple gives reading of temperature on its own tips. This specific configuration of interest can be ascertained from the readings of temperature with change in concentration of methylene blue solution (the temperature reading decreases with an increase in concentration (for direct incident) while the reading increases with an increase in concentration (not incident directly).
